# Bidirectional two-sample Mendelian randomization study of association between smoking initiation and atrial fibrillation

**DOI:** 10.18332/tid/189380

**Published:** 2024-06-19

**Authors:** Ziyang Wu, Chengchun Tang, Dong Wang

**Affiliations:** 1 School of Medicine, Southeast University, Nanjing, China; 2 Department of Cardiology, School of Medicine, Southeast University, Zhongda Hospital, Nanjing, China

**Keywords:** smoking initiation, atrial fibrillation, two-sample Mendelian randomization

## Abstract

**INTRODUCTION:**

The relationship between smoking and heart disease has been frequently reported. Therefore, we aimed to explore the association between smoking initiation and atrial fibrillation.

**METHODS:**

Genetic association data pertaining to smoking initiation and atrial fibrillation were obtained from genome-wide association studies (GWAS). Phenotypically related single nucleotide polymorphisms (SNPs) were selected as instrumental variables. Inverse-variance weighted (IVW), weighted median, Mendelian randomization (MR), Egger regression, simple mode, and weighted mode methods were employed to perform the MR study. The association between smoking initiation and atrial fibrillation was evaluated using odds ratios (OR) and 95% confidence intervals (CI). Cochran’s Q test was employed to assess heterogeneity among instrumental variables, utilizing the IVW and MR-Egger methods. The Egger-intercept method was employed to test for horizontal pleiotropy, and the ‘leave-one-out’ method was utilized for sensitivity analysis.

**RESULTS:**

The MR results for the effect of smoking initiation on atrial fibrillation (IVW, OR=1.11; 95% CI: 1.02–1.20, p=0.013) supported an association between smoking initiation and an increased likelihood of atrial fibrillation. In total, 85 SNPs were extracted from the GWAS pooled data as instrumental variables. The MR-Egger method indicated an intercept close to 0 (Egger intercept= -0.005, p=0.371), suggesting no horizontal pleiotropy in the selected instrumental variables. The ‘leave-one-out’ sensitivity analysis demonstrated that the results were robust and that no instrumental variables significantly influenced the results. Reverse MR analysis indicated no effect of atrial fibrillation on smoking initiation (IVW, OR=1.00; 95% CI: 0.99–1.02, p=0.684).

**CONCLUSIONS:**

Smoking initiation has a significant impact on atrial fibrillation. However, atrial fibrillation did not influence smoking initiation. This study provides novel insights into the genetic relationships between smoking initiation and atrial fibrillation.

## INTRODUCTION

Smoking is a well-known risk factor for cardiovascular disease and has been extensively studied for its detrimental effects on blood vessels, cardiac structure, and rhythm^1^. Research conducted by the American Heart Association has shown that smoking causes structural damage to arterial walls, resulting in premature aging of blood vessels by up to 10 years^2^. Additionally, smoking increases the risk of heart attacks by promoting embolism and coronary artery disease. Smoking cessation has been included in routine treatment plans in clinical practice due to proposed insights from studies, such as increasing platelet sensitivity, reducing oxygen supply, reducing the ability of the myocardium to utilize oxygen, increasing cardiac oxygen consumption, and other mechanisms^3^.

Atrial fibrillation is a re-entrant arrhythmia characterized by irregular and rapid contractions of the atrial muscle cells. It can manifest with various clinical symptoms, including palpitations, dizziness, chest tightness, fatigue, and a general sense of dullness, though some individuals may be asymptomatic. Atrial fibrillation can lead to serious complications, including stroke and heart failure. It frequently occurs in patients with underlying conditions, such as coronary heart disease, hyperthyroidism, and hypertension^4^. It is also associated with unhealthy lifestyle habits, including smoking and drinking. Research into the relationship between smoking initiation and cessation and the incidence of atrial fibrillation is ongoing, with studies yielding conflicting results^5,6^.

MR technique uses genetic variation as an instrumental variable (IV) to assess whether exposure factors affect outcomes. This technique avoids the influence of confounders and reverse associations on correlation effects, thus minimizing bias. Genetic variation is a stable exposure factor over time and is not influenced by external environmental or social factors^7^. This study collected and analyzed published data using bidirectional TSMR to investigate the existence of the relationship between smoking initiation and atrial fibrillation.

## METHODS

### Data sources

This is a secondary dataset analysis of the GWAS datasets. GWAS datasets were obtained from the IEU OpenGWAS project (https://gwas.mrcieu.ac.uk). The GWAS dataset for smoking initiation was derived from GWAS analysis and included 311629 cases and 321173 controls of European ancestry^8^. Smoking initiation phenotypes included age of initiation of regular smoking, a binary phenotype indicating whether an individual had ever smoked regularly, and heaviness of smoking measured with cigarettes per day. The GWAS dataset for atrial fibrillation was derived from another analysis and included 60620 cases and 970216 controls of European ancestry^9^. Atrial fibrillation phenotypes included the duration of long-lasting atrial fibrillation, the age of onset of atrial fibrillation, and atrial fibrillation caused by other diseases. These phenotypes are mainly displayed through electrocardiogram-related data.

### Screening of IVs

The genome-wide association study data identified SNPs with exposure variables (p<5.0×10^-8^). To ensure the independence of SNPs and eliminate the confounding effect of linkage disequilibrium (LD), clumping was conducted using the TwoSampleMR package in R software. The parameters were set to r^2^ <0.001 and a distance threshold of 10000 kb. In order to ascertain that the selected IVs satisfy the independence assumption, the remaining SNPs were subjected to an association test with other phenotypes using PhenoScanner (http://www.phenoscanner.medschl.cam.ac.uk). The association hypothesis was further tested by calculating the F-statistic to assess the presence of weak IV bias in the selected IVs. The absence of weak IV bias was confirmed by an F-statistic value >10, where:

F = R^2^ (N-2)/ (1-R^2^)^10^.

### Research design

MR studies must satisfy three core assumptions^11^: association, independence, and exclusivity. These assumptions are as follows: 1) the IVs must be strongly associated with the exposure factor; 2) the IVs should not be associated with any confounders that are associated with the exposure or outcome; and 3) the IVs can only influence the outcome variable through the exposure factor. Supplementary file Figure 1 illustrates the principles of MR.

### Statistical analysis

To ensure that the effect of SNPs on exposure and outcome corresponded to the same alleles, the summary statistics of the exposure and outcome datasets were harmonized. Associations were inferred using TSMR analyses involving the IVW method, weighted median, MR Egger regression, and simple and weighted mode methods. OR and 95% CI were calculated for the association between smoking initiation and atrial fibrillation. The IVW method was primarily used for Mendelian randomization. The IVW method combined the Wald ratio estimates of the effects of different SNPs when each genetic variation met the IV hypothesis. This provided a consistent estimate of the effect of exposure on the outcome. The reliability of the IVW method’s results is highest when there is no horizontal pleiotropy of the IVs. The weighted median provided a consistent estimate of the effect when at least half of the SNPs were effective IVs. MR-Egger regression confirmed horizontal pleiotropy for IVs, with its intercept representing the effect estimate. MR-Egger regression is based on the assumption of instrument strength independent of direct effect (InSIDE). When horizontal pleiotropy is present in the IVs, the MR-Egger regression can still provide an unbiased estimate of the association. To improve the accuracy of the results, compared to the MR-Egger method, we performed complementary analyses using the weighted median method and the simple mode and weighted mode. To detect and correct horizontal pleiotropy by removing outliers, we used the Mendelian randomization pleiotropy residual sum and outlier (MR-PRESSO) test^12^. Statistical analysis was performed using R (version 4.3.1) and R packages (TwoSampleMR and MR-PRESSO). The test level α was 0.05 (p<0.05), for the difference to be statistically significant. All tests were two-tailed.

## RESULTS

### Effects of smoking initiation on atrial fibrillation

**Figure 1:**
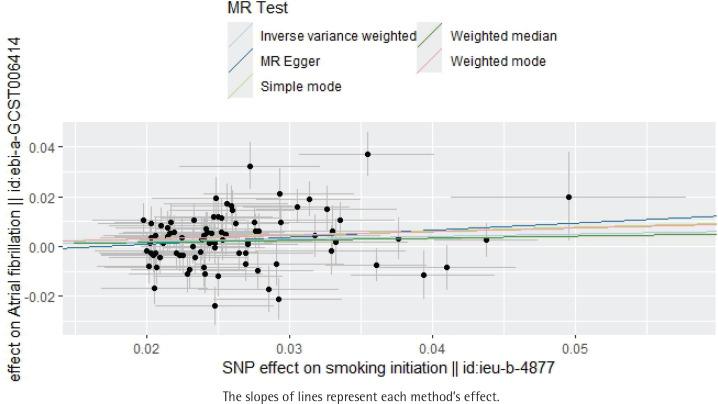
Scatter plot of the effect of smoking initiation on atrial fibrillation, IEU OpenGWAS 2018–2019 (N=372249)

Smoking initiation was the exposure factor, and atrial fibrillation was the outcome variable. The screening criteria yielded a total of 85 SNPs included in the study as IVs. Each F-statistic associated with instrumental exposure was >10, effectively eliminating weak IVs from biasing the results (Supplementary file Table 1). The horizontal pleiotropy test results, obtained using the Egger-intercept method, resulted in p=0.371 (Egger intercept= -0.005), indicating that the IVs did not significantly affect the outcome through pathways other than exposure (Table 1, Figure 1).

**Table 1:** Heterogeneity test and horizontal pleiotropy test, IEU OpenGWAS 2018–2019 (N=372249)

*Exposure*	*Outcome*	*Heterogeneity test (MR Egger)*			*Heterogeneity test (IVW)*			*Horizontal pleiotropy test (MR Egger)*	
		*Cochran’s Q*	*Q df*	*p*	*Cochran’s Q*	*Q df*	*p*	*Intercept*	*p*
Smoking initiation	Atrial fibrillation	138.505	83	<0.001	139.858	84	<0.001	-0.005	0.371
Atrial fibrillation	Smoking initiation	230.085	107	<0.001	231.846	108	<0.001	-0.001	0.367

IVW: inverse-variance weighted.

**Figure 2:**
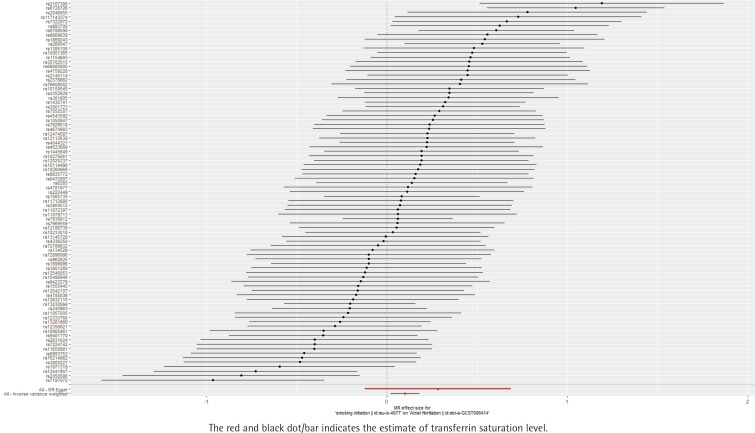
Forest plot of the effect of smoking initiation on atrial fibrillation, IEU OpenGWAS 2018–2019 (N=372249)

Without horizontal pleiotropy, the results of the TSMR were analyzed using the IVW method of the random-effects model. The IVW method suggested that the presence of smoking initiation was associated with a 1.11-fold increased likelihood of atrial fibrillation (IVW result: OR=1.11; 95% CI: 1.02–1.20, p=0.013). The following results were obtained: MR Egger, OR=1.33; 95% CI: 0.89–1.99, p=0.175; weighted median, OR=1.09; 95% CI: 0.99–1.12, p=0.094; simple mode, OR=1.17; 95% CI: 0.90–1.52, p=0.248; and weighted mode, OR=1.16; 95% CI: 0.92–1.46, p=0.224 (Table 2, Figures 1 and 2).

**Table 2:** Mendelian randomization analysis of association between smoking initiation and the risk of atrial fibrillation, IEU OpenGWAS 2018–2019 (N=372249)

*Methods*	*SNPs*	*β*	*SE*	*OR (95% CI)*	*p*
MR Egger	85	0.283	0.207	1.33 (0.89–1.99)	0.175
Weighted median	85	0.082	0.049	1.09 (0.99–1.20)	0.094
Inverse variance weighted	85	0.100	0.040	1.11 (1.02–1.20)	0.013
Simple mode	85	0.156	0.135	1.17 (0.90–1.52)	0.248
Weighted mode	85	0.145	0.118	1.16 (0.92–1.46)	0.224

MR: mendelian randomization. SNPs: single nucleotide polymorphisms. SE: standard error. OR and 95% CI were calculated using R 4.3.1 software and TwoSampleMR R packages: OR< generated odds ratios (MR results).

**Figure 3:**
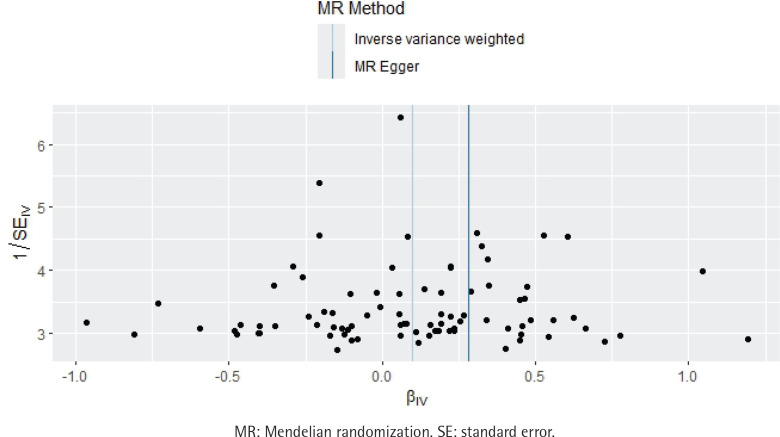
Funnel plot of the effect of smoking initiation on atrial fibrillation, IEU OpenGWAS 2018–2019 (N=372249)

The funnel plot indicates that all SNPs were largely symmetrical, suggesting minor differences between IVs. Although Cochran’s Q test indicated heterogeneity (MR-Egger regression: Cochran’s Q=138.51, p<0.001; IVW: Cochran’s Q=139.86, p<0.001), the results of the random-effects IVW method indicated that there was indeed an association between smoking initiation and AF (Table 1, Figure 3). Furthermore, the MR-PRESSO method consistently yielded estimates before and after outlier correction.

**Figure 4:**
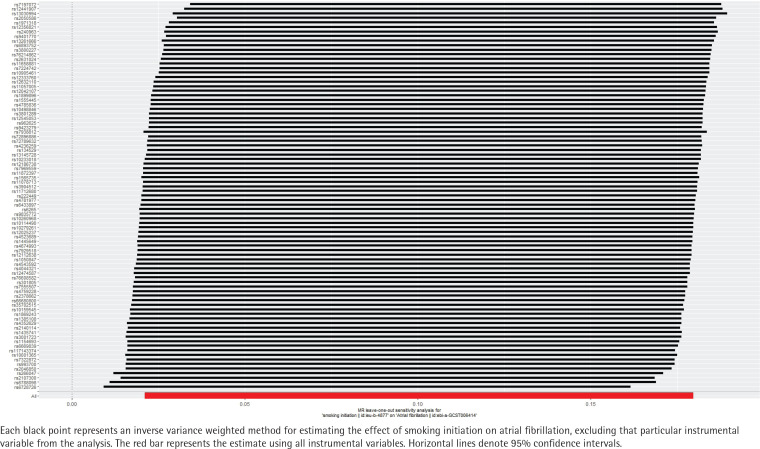
Sensitivity analysis of the effect of smoking initiation on atrial fibrillation, IEU OpenGWAS 2018–2019 (N=372249)

The leave-one-out sensitivity analysis demonstrated that excluding each SNP in turn produced IVW analysis results comparable to those including all SNPs. Furthermore, no SNPs were identified as having a substantial impact on the association estimates. No variants strongly associated with the potential confounders were found in the instrumental variables using PhenoScanner V2, indicating that the TSMR analysis results were robust (Figure 4).

### Reverse TSMR analysis

In reverse TSMR, atrial fibrillation was the exposure factor, and smoking initiation was the outcome variable. A total of 109 SNPs were included in this study. All SNPs were found to be significantly associated with atrial fibrillation (p<5×10^-8^), with F-statistics exceeding 10 (Supplementary file Table 2). The results of the horizontal pleiotropy test, with p>0.05 (Egger intercept = -0.001, p=0.367) (Table 1), indicates no horizontal pleiotropy for the IVs. The following MR results were obtained: IVW, OR=1.00; 95% CI: 0.99–1.02, p=0.684; MR Egger, OR=1.02; 95% CI: 0.99–1.05, p=0.326; weighted median, OR=1.02; 95% CI: 1.00–1.04, p=0.090; simple mode, OR=1.01; 95% CI: 0.96–1.05, p=0.825; and weighted mode, OR=1.01; 95% CI: 0.99–1.03, p=0.392 (Table 3, Supplementary file Figures 2 and 3). The results of the heterogeneity test indicate the presence of significant heterogeneity among the selected IVs (MR Egger regression: Cochran’s Q=230.09, p<0.001; IVW: Cochran’s Q=231.85, p<0.001) (Table 1, Supplementary file Figure 4). Furthermore, no outliers or instances of directed pleiotropy were identified in the IVs as analyzed by MR-PRESSO. The leave-one-out sensitivity analysis demonstrated that individual SNP did not influence the genetic results (Supplementary file Figure 5).

**Table 3:** Mendelian randomization analysis of association between atrial fibrillation and smoking initiation, IEU OpenGWAS 2018–2019 (N=372249)

*Methods*	*SNPs*	*β*	*SE*	*OR (95%CI)*	*p*
MR Egger	109	0.015	0.015	1.02 (0.99–1.05)	0.326
Weighted median	109	0.016	0.009	1.02 (1.00–1.04)	0.090
Inverse variance weighted	109	0.003	0.008	1.00 (0.99–1.02)	0.684
Simple mode	109	0.005	0.021	1.01 (0.96–1.05)	0.825
Weighted mode	109	0.010	0.011	1.01 (0.99–1.03)	0.392

MR: mendelian randomization. SNPs: single nucleotide polymorphisms. SE: standard error. OR and 95% CI were calculated using R 4.3.1 software and TwoSampleMR R packages: OR< generated odds ratios (MR results).

## DISCUSSION

This study used the bidirectional TSMR method to analyze published GWAS datasets and determine whether a bidirectional association exists between smoking initiation and atrial fibrillation. Our results supported an association between smoking initiation and an increased likelihood of atrial fibrillation. However, our results did not support an association between atrial fibrillation and an increased frequency of smoking initiation. In the sensitivity analysis, the MR results were robust and reliable.

Smoking is a well-established risk factor for cardiovascular disease, which can lead to serious health issues such as heart attacks and strokes. It is widely acknowledged that tobacco components are associated with atrial fibrillation, an irregular heartbeat that can be life-threatening if left untreated. Nicotine, a primary component in tobacco, stimulates sympathetic nervous system transmission, resulting in increased heart rate and blood pressure^13,14^. Smoking initiation can elevate plasma catecholamine concentration, which may have detrimental effects on the cardiovascular system^15^. Additionally, tobacco contains carbon monoxide, which is highly toxic to the heart. Carbon monoxide diminishes hemoglobin’s oxygen-carrying capacity, thereby reducing oxygen release to body tissues^16^. This can lead to a decrease in cardiac exercise tolerance and an increased risk of heart disease.

Smoking initiation is linked to an elevated risk of atherosclerosis, the buildup of plaque in arteries^17^. This process is caused by more than 7000 chemicals found in cigarettes, such as tar, nicotine, and carbon monoxide. These chemicals can cause cardiovascular disease by increasing heart rate and myocardial contractility, promoting inflammation, causing endothelial damage and thrombosis, and lowering serum high-density lipoprotein cholesterol levels^18-20^. Inflammation plays an important role in the development of atrial fibrillation^21^. Endothelial damage is particularly concerning as it can lead to the formation of blood clots, which can result in heart attacks or strokes. Smoking initiation causes inflammation, which promotes the development of atherosclerosis by leading to the accumulation of immune cells on the arterial wall^22^. This accumulation narrows the artery and reduces blood flow.

In summary, smoking initiation has a significant impact on cardiovascular disease. It is important to understand the risks associated with tobacco use. Cigarettes contain various chemicals that can have adverse effects on the cardiovascular system. Smoking initiation can have negative effects on health, including the development of atherosclerosis and arrhythmia, as well as an increased risk of sudden cardiac death^18,23^. Smoking cessation is the best way to reduce these risks and improve overall cardiovascular health.

Previous studies often had several common issues. The first one is that their assessment of the severity of smoking depended on self-report ^24,25^, which may be subject to inaccuracies and misclassification, potentially leading to biased estimates. It is important to note that self-reported data can be influenced by social desirability bias, recall bias, and other factors that may affect the accuracy of the results. Our research precisely avoids potential biases. Secondly, they frequently used plasma cotinine measurement to adjust their research findings ^26-28^. However, this method is susceptible to various factors and may not be entirely accurate. As a result, the conclusions drawn from this correction method may not be entirely convincing. A new and more effective correction method may solve the existing problems. Thirdly, they did not solely examine the relationship between smoking initiation and atrial fibrillation but conducted a comprehensive analysis of various risk factors, including age, gender, smoking, alcohol consumption, and medication^29,30^. While appropriate measures were taken to minimize correlation, it may still exist. The research presented in this study avoids the issue at the genetic level.

MR uses genetic variation to estimate the health consequences of phenotypes affected by these genetic variations. This epidemiological approach infers the association between exposure factors and outcome variables using genetic variation^31^. MR provides a method to investigate associations without the typical biases inherent in observational epidemiological studies, such as reverse association and potential confounders^32^. However, larger GWAS studies will be necessary in the future.

### Limitations

This study has some limitations. First, the MR results were based on the GWAS dataset derived from individuals with European ancestry, preventing the extrapolation of our results to other ethnicities. Further research is needed to determine whether a relationship exists in other populations. Second, genetic polymorphisms may lead to correlations between the SNPs used for analysis and other traits, resulting in confounding bias that can affect inference. Third, the strength of the IV depends on the sample size of the GWAS. Conducting a large-scale GWAS is necessary to identify additional genetic variations for MR. Fourth, we could not address potential pleiotropy that may have remained undetected, which may impact the perceived credibility of our results. Fifth, the proportion of smokers among males is significantly higher than that among females. However, the data utilized in this study were derived from public databases, making it unsuitable for subgroup analysis of specific factors such as gender. Finally, studies focusing on smoking initiation and atrial fibrillation utilized in this TSMR analysis may have included the same participants. However, we were unable to determine the number of overlapping participants in the datasets, limiting our ability to mitigate potential bias due to sample overlap.

## CONCLUSIONS

Bidirectional TSMR analysis supported the association between smoking initiation and an increased likelihood of atrial fibrillation. Still, it did not support an association between atrial fibrillation and increased smoking initiation frequency. However, due to the study’s limitations, further research is necessary.

## Supplementary Material



## Data Availability

All data generated or analyzed during this study are included in this published article and its Supplementary file.

## References

[1] Jiang X, Alnoud MAH, Ali H, et al. Heartfelt living: deciphering the link between lifestyle choices and cardiovascular vitality. Curr Probl Cardiol. 2024;49(3):102397. doi:10.1016/j.cpcardiol.2024.102397 38232921

[2] Martin SS, Aday AW, Almarzooq ZI, et al. 2024 Heart disease and stroke statistics: a report of US and global data from the American Heart Association. Circulation. 2024;149(8):e347-e913. doi:10.1161/CIR.0000000000001209 38264914 PMC12146881

[3] McCaughey CJ, Murphy G, Jones J, Mirza KB, Hensey M. Safety and efficacy of e-cigarettes in those with atherosclerotic disease: a review. Open Heart. 2023;10(2):e002341. doi:10.1136/openhrt-2023-002341 38065586 PMC10711928

[4] Shantsila E, Choi EK, Lane DA, Joung B, Lip GYH. Atrial fibrillation: comorbidities, lifestyle, and patient factors. Lancet Reg Health Eur. 2024;37:100784. doi:10.1016/j.lanepe.2023.100784 38362547 PMC10866737

[5] Albertsen IE, Overvad TF, Lip GY, Larsen TB. Smoking, atrial fibrillation, and ischemic stroke: a confluence of epidemics. Curr Opin Cardiol. 2015;30(5):512-517. doi:10.1097/HCO.0000000000000205 26172213

[6] Lu Y, Guo Y, Lin H, Wang Z, Zheng L. Genetically determined tobacco and alcohol use and risk of atrial fibrillation. BMC Med Genomics. 2021;14(1):73. doi:10.1186/s12920-021-00915-0 33750369 PMC7944892

[7] Birney E. Mendelian Randomization. Cold Spring Harb Perspect Med. 2022;12(4):a041302. doi:10.1101/cshperspect.a041302 34872952 PMC9121891

[8] Liu M, Jiang Y, Wedow R, et al. Association studies of up to 1.2 million individuals yield new insights into the genetic etiology of tobacco and alcohol use. Nat Genet. 2019;51(2):237-244. doi:10.1038/s41588-018-0307-5 30643251 PMC6358542

[9] Nielsen JB, Thorolfsdottir RB, Fritsche LG, et al. Biobank-driven genomic discovery yields new insight into atrial fibrillation biology. Nat Genet. 2018;50(9):1234-1239. doi:10.1038/s41588-018-0171-3 30061737 PMC6530775

[10] Guo HY, Wang W, Peng H, Yuan H. Bidirectional two-sample Mendelian randomization study of causality between rheumatoid arthritis and myocardial infarction. Front Immunol. 2022;13:1017444. doi:10.3389/fimmu.2022.1017444 36532051 PMC9755576

[11] An L, Ren X, Pan Y, et al. IFN-γ, SCF, MIP1b and IL-16 were associated with risk of diabetic nephropathy: a Mendelian randomization study. Diabetes Metab Syndr Obes. 2024;17:851-856. doi:10.2147/DMSO.S452227 38410634 PMC10895979

[12] Hou Y, Si K, Yang J, et al. Association between regulatory T cells and ischemic heart disease: a Mendelian randomization study. J Thorac Dis. 2024;16(1):564-572. doi:10.21037/jtd-23-1790 38410592 PMC10894418

[13] Dorotheo EU, Arora M, Banerjee A, et al. Nicotine and cardiovascular health: when poison is addictive - a WHF Policy Brief. Glob Heart. 2024;19(1):14. doi:10.5334/gh.1292 38312998 PMC10836189

[14] Del Calvo G, Pollard CM, Baggio Lopez T, Borges JI, Suster MS, Lymperopoulos A. Nicotine diminishes alpha2-adrenergic receptor-dependent protection against oxidative stress in H9c2 cardiomyocytes. Drug Des Devel Ther. 2024;18:71-80. doi:10.2147/DDDT.S432453 PMC10790636 38229917

[15] Jones CA, Wallace MJ, Bandaru P, Woodbury ED, Mohler PJ, Wold LE. E-cigarettes and arrhythmogenesis: a comprehensive review of pre-clinical studies and their clinical implications. Cardiovasc Res. 2023;119(12):2157-2164. doi:10.1093/cvr/cvad113 37517059 PMC10578912

[16] Jiang M, Yu CH, Xu Z, Qin Z. Binding of carbon monoxide to hemoglobin in an oxygen environment: force field development for molecular dynamics. J Chem Theory Comput. 2024;20(10):4229-4238. doi:10.1021/acs.jctc.4c00029 38400860 PMC11137813

[17] Cook SH, Wood EP, Stein JH, McClelland RL. Discrimination, smoking, and cardiovascular disease risk: a moderated mediation analysis with MESA. J Am Heart Assoc. 2024;13(5):e032659. doi:10.1161/JAHA.123.032659 38390806 PMC10944061

[18] Da H, Yang R, Liang J, et al. Association of a low-inflammatory diet with survival among adults: the role of cardiometabolic diseases and lifestyle. Clin Nutr. 2024;43(4):943-950. doi:10.1016/j.clnu.2024.02.022 38422952

[19] Tang S, Meng J, Zhao X, Sun W. Trends of ischemic heart disease mortality attributable to smoking in the five countries with the highest number of smokers during 1990-2019: an age-period-cohort analysis. Arch Med Sci. 2024;20(1):43-53. doi:10.5114/aoms/182886 38414476 PMC10895949

[20] Chen HY, Li SC, Chen LF, Wang W, Wang Y, Yan XW. The effects of cigarette smoking and smoking cessation on high-density lipoprotein functions: implications for coronary artery disease. Ann Clin Biochem. 2019;56(1):100-111. doi:10.1177/0004563218788386 29961342

[21] Ajoolabady A, Nattel S, Lip GYH, Ren J. Inflammasome signaling in atrial fibrillation: JACC state-of-the-art review. J Am Coll Cardiol. 2022;79(23):2349-2366. doi:10.1016/j.jacc.2022.03.379 35680186 PMC8972346

[22] Wang Z, Xie J, Wu C, Xiao G. Correlation between smoking and passive smoking with multiple sclerosis and the underlying molecular mechanisms. Med Sci Monit. 2019;25:893-902. doi:10.12659/MSM.912863 30703074 PMC6367889

[23] Strayer SM, Barnhardt A, Rollins LK, et al. Assessing efficacy of a web-based smoking cessation tool - QuitAdvisorMD: protocol for a practice-based, clustered, randomized control trial. Contemp Clin Trials Commun. 2024;38:101253. doi:10.1016/j.conctc.2023.101253 38404651 PMC10884820

[24] Qian Y, Tan JB, Wang T, et al. Quality appraisal and descriptive analysis of clinical practice guidelines for self-managed non-pharmacological interventions of cardiovascular diseases: a systematic review. J Transl Med. 2024;22(1):215. doi:10.1186/s12967-024-04959-5 38424641 PMC10903016

[25] Leszto K, Frąk W, Kurciński S, et al. Associations of dietary and lifestyle components with atrial fibrillation. Nutrients. 2024;16(3):456. doi:10.3390/nu16030456 38337740 PMC10856828

[26] Li C, Guo Y, Duan K, et al. Changes in biomarkers of exposure and withdrawal symptom among Chinese adult smokers after completely or partially switching from combustible cigarettes to an electronic nicotine delivery system. Intern Emerg Med. 2024;19(3):669-679. doi:10.1007/s11739-023-03518-y 38316693

[27] Haervig KK, Petersen KU, Dornfeldt MM, et al. Paternal pre-conceptional smoking and semen quality in the adult son. Andrology. 2023:1-7. doi:10.1111/andr.13550 PMC11635548 37885366

[28] Zhang L, Zhu Y, Meng X, et al. Smoking, immunity, and cardiovascular prognosis: a study of plasma IgE concentration in patients with acute myocardial infarction. Front Cardiovasc Med. 2023;10:1174081. doi:10.3389/fcvm.2023.1174081 37731521 PMC10508960

[29] Mohammadi S, Paryad E, Khanghah AG, Leili EK, Noveiri MJS. Investigate the relationship between obstructive sleep apnea and cardiac arrhythmia after CABG surgery. BMC Cardiovasc Disord. 2024;24(1):64. doi:10.1186/s12872-023-03694-x 38263001 PMC10804646

[30] Yang E, Heckbert SR, Ding J, et al. Prevalence of subclinical atrial fibrillation in heart failure with preserved ejection fraction. JACC Heart Fail. 2024;12(3):492-504. doi:10.1016/j.jchf.2023.09.023 37999661

[31] Dixon P, Martin RM, Harrison S. Causal estimation of long-term intervention cost-effectiveness using genetic instrumental variables: an application to cancer. Med Decis Making. 2024;44(3):283-295. doi:10.1177/0272989X241232607 38426435 PMC10988994

[32] Millard LAC, Davey Smith G, Tilling K. Using the global randomization test as a Mendelian randomization falsification test for the exclusion restriction assumption. Eur J Epidemiol. 2024:1-13. doi:10.1007/s10654-024-01097-6 PMC11410989 38421485

