# Supplementary material for: Bidirectional two-sample Mendelian randomization study of association between smoking initiation and atrial fibrillation
Source: Tob Induc Dis. 2024 Jun 19;22:10.18332/tid/189380. doi: 10.18332/tid/189380 (PMC11186308; doi:10.18332/tid/189380)
Supplement: Supplementary file 1 [file TID-22-113-s1.pdf]

Supplementary Figure 1 Principles of two-sample mendelian randomization, IEU OpenGWAS 2018-2019 (N=372,249)

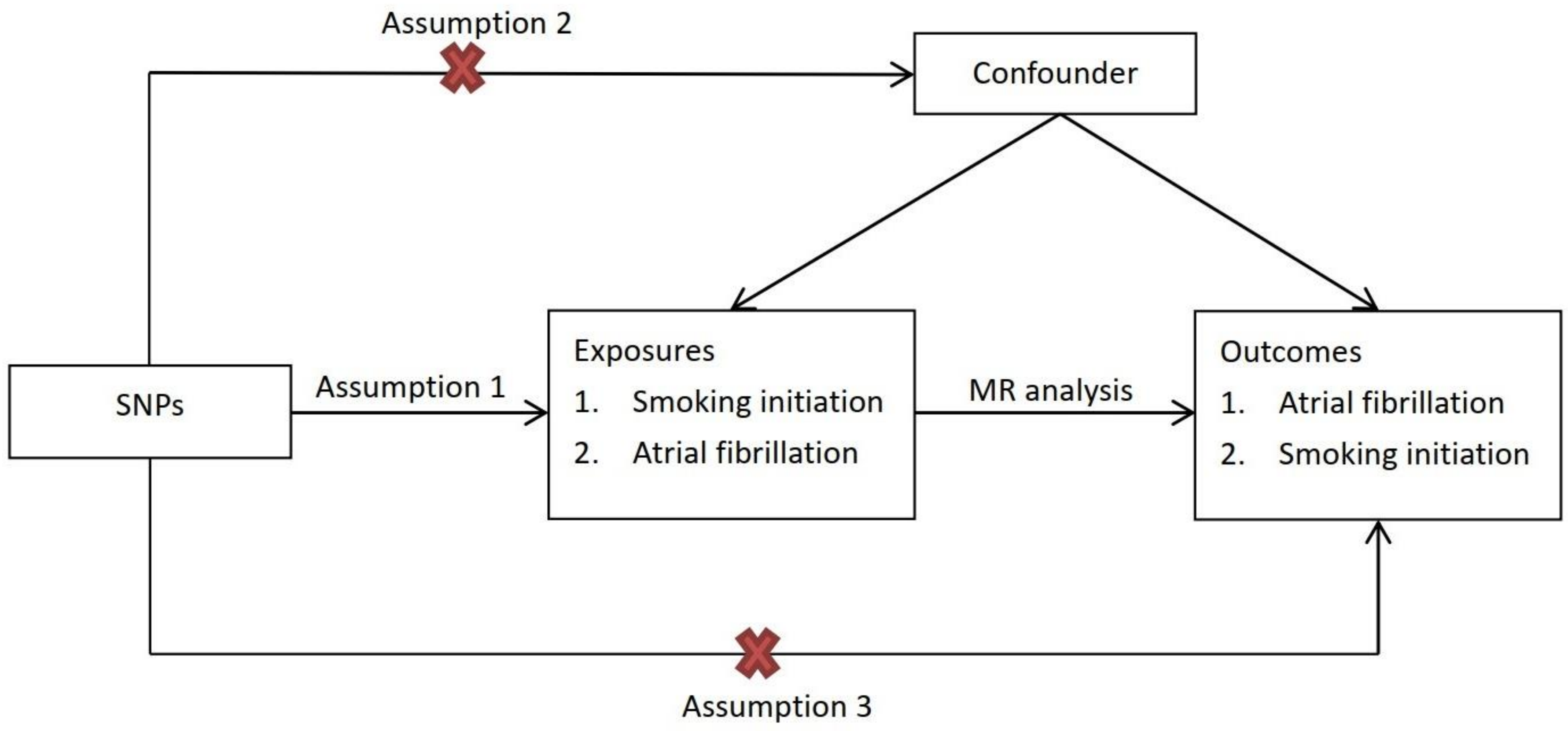

SNPs, single nucleotide polymorphisms; MR, mendelian randomization; MR analysis was performed using inverse-variance weighted, weighted median, MR Egger regression, simple mode, and weighted mode methods.

Supplementary Figure 2 Scatter plot of the effect of atrial fibrillation on smoking initiation, IEU OpenGWAS 2018-2019 (N=372,249)

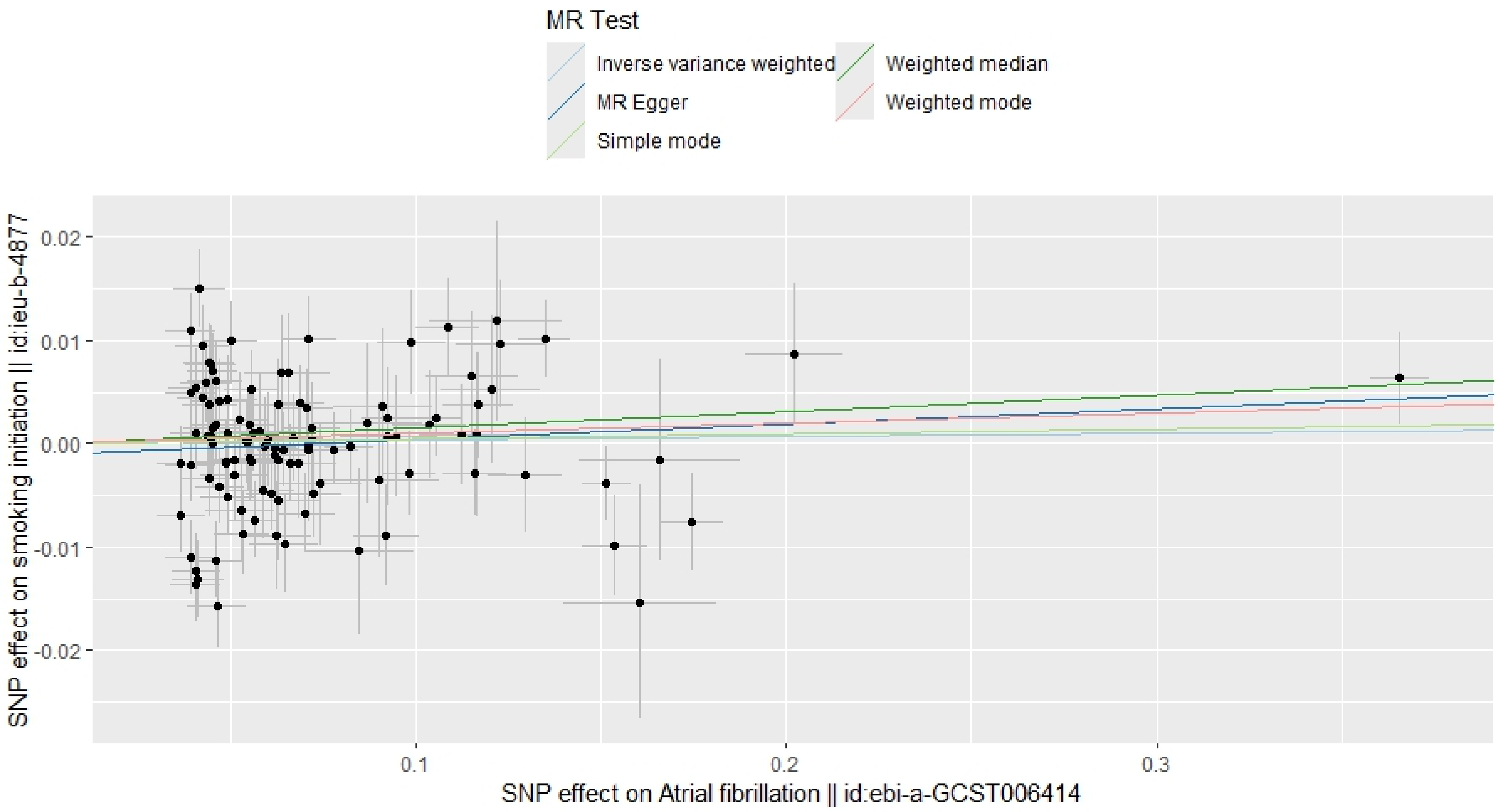

The slopes of lines represent the effect of each method, respectively.

Supplementary Figure 3 Forest plot of the effect of atrial fibrillation on smoking initiation, IEU OpenGWAS 2018-2019 (N=372,249)

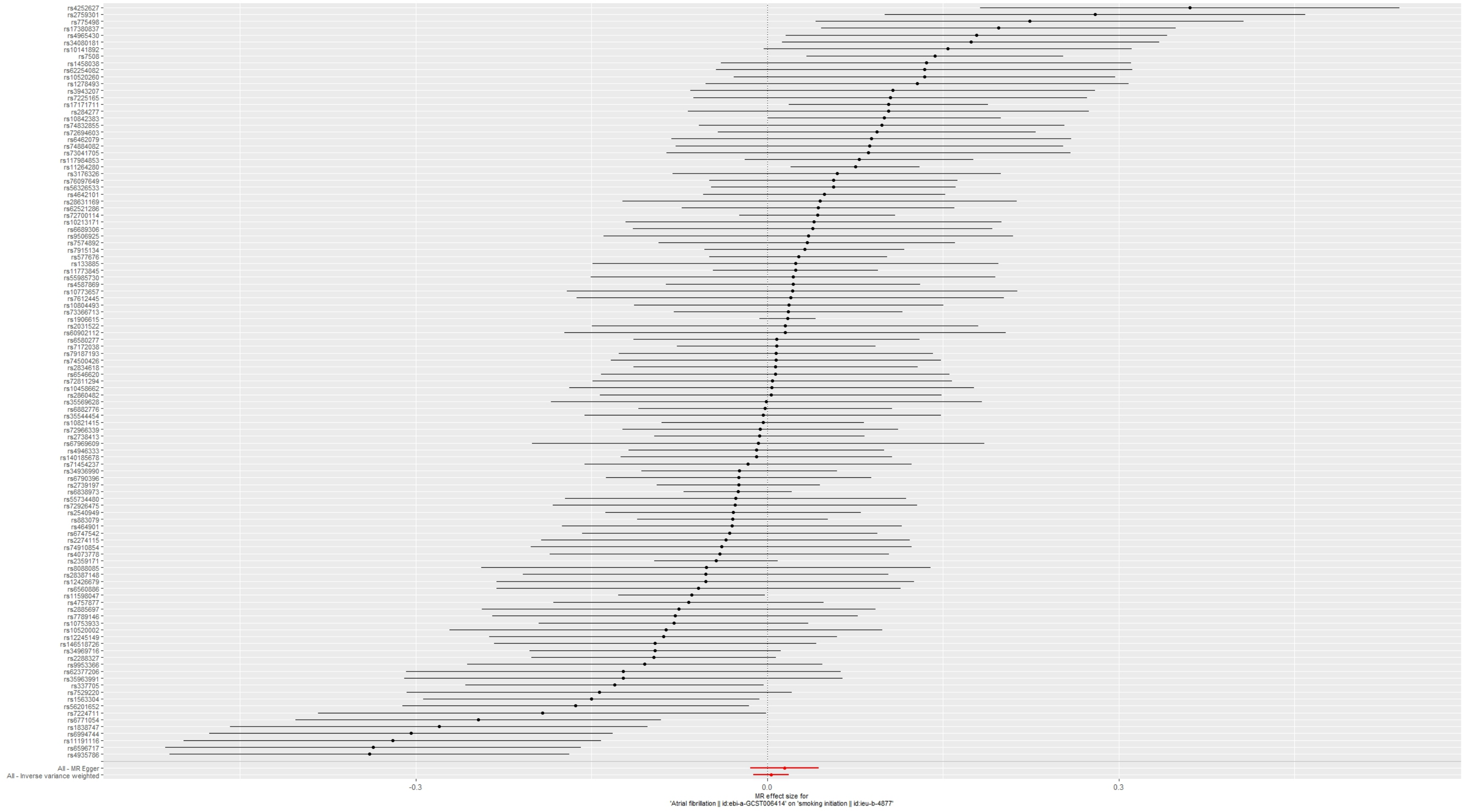

The red and black dots/bars indicate the estimate of transferrin saturation level.

Supplementary Figure 4 Funnel plot of the effect of atrial fibrillation on smoking initiation, IEU OpenGWAS 2018-2019 (N=372,249)

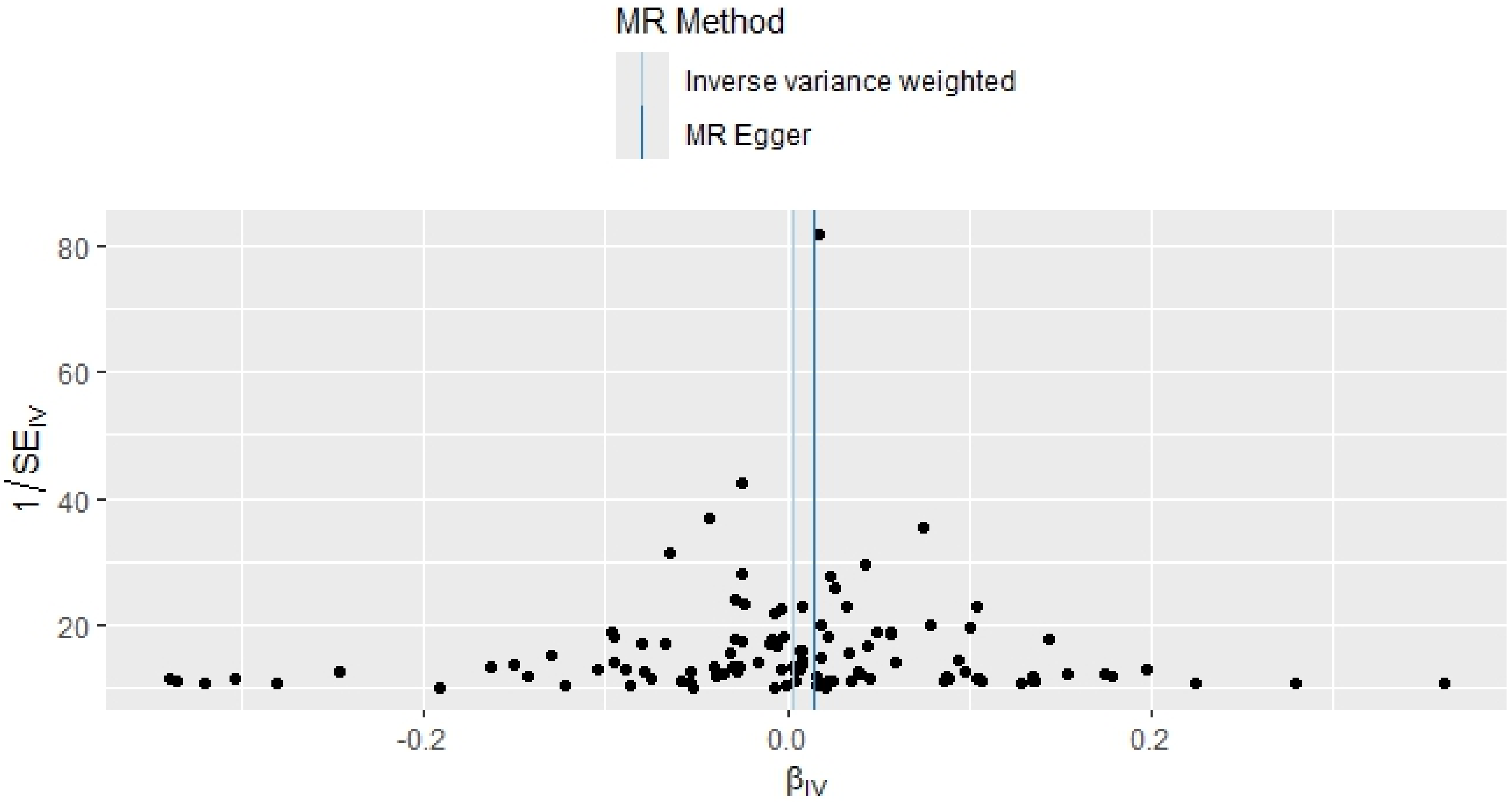

MR, mendelian randomization; SE, standard error.

Supplementary Figure 5 Sensitivity analysis of the effect of atrial fibrillation on smoking initiation, IEU OpenGWAS 2018-2019 (N=372,249)

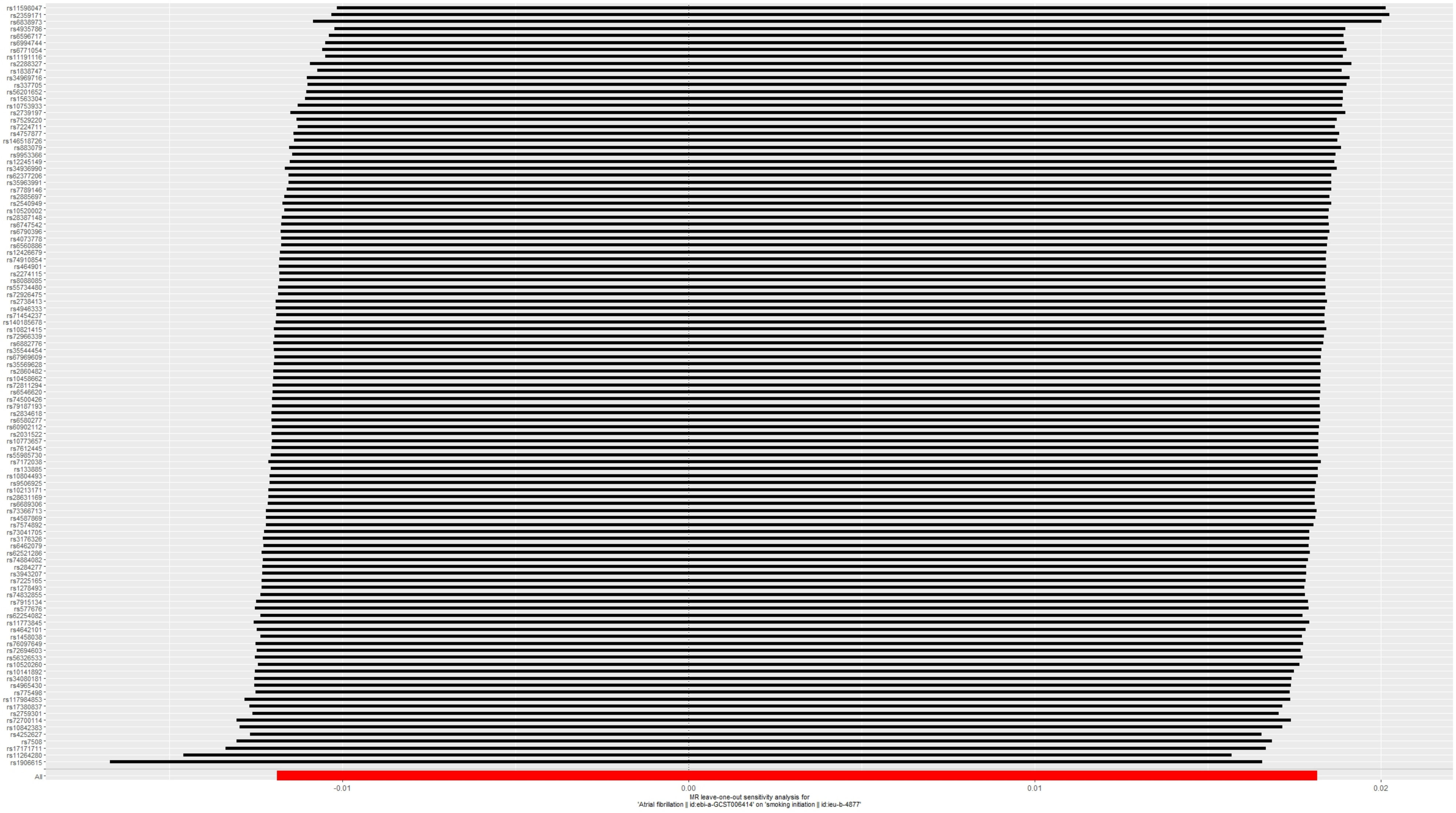

Each black point represents an inverse variance weighted method for estimating the effect of atrial fibrillation on smoking initiation, excluding that particular instrumental variable from the analysis. Red bar represents the estimate using all instrumental variables. Horizontal lines denote 95% confidence intervals.

Supplementary Table 1 Summary statistics for the instrumental variables associated with smoking initiation and atrial fibrillation, IEU OpenGWAS 2018-2019 (N=372,249)

SNPs	chromosome	position	effect allele	other allele	EAF	smoking initiation				atrial fibrillation		
						beta	SE	p	sample size	beta	SE	p
rs10001365	4	1.48E+08	A	G	0.397	-0.025	0.004	6.65E-12	632802	-0.012	0.007	0.080
rs10114490	9	11070165	A	G	0.189	-0.026	0.005	1.81E-08	632802	-0.005	0.008	0.578
rs10159545	10	21766969	G	C	0.351	0.026	0.004	1.84E-12	632802	0.009	0.007	0.197
rs10233018	7	1.18E+08	G	A	0.523	0.027	0.004	2.75E-14	632802	0.001	0.007	0.899
rs10260968	7	1889773	A	G	0.578	-0.020	0.004	1.75E-08	632802	-0.004	0.007	0.601
rs10279261	7	1.34E+08	A	G	0.609	-0.021	0.004	5.00E-09	632802	-0.004	0.007	0.554
rs10498846	6	67405337	T	C	0.508	0.021	0.004	6.62E-09	632802	-0.003	0.007	0.690
rs1050847	16	87443734	T	C	0.567	-0.022	0.004	1.67E-09	632802	-0.006	0.007	0.415
rs10905461	10	8803551	C	T	0.745	-0.024	0.004	7.35E-09	632802	0.008	0.008	0.281
rs11057005	12	16748721	G	A	0.441	-0.021	0.004	4.85E-09	632802	0.005	0.007	0.502
rs11078713	17	7795972	G	A	0.413	-0.020	0.004	2.23E-08	632802	-0.001	0.007	0.855
rs1154693	3	1.18E+08	G	A	0.822	0.033	0.005	3.12E-11	632802	0.015	0.009	0.101
rs11658881	17	2072949	G	A	0.415	0.020	0.004	2.43E-08	632802	-0.008	0.007	0.229
rs11712680	3	75009019	C	A	0.187	-0.027	0.005	3.51E-09	632802	-0.002	0.009	0.807
rs117143374	21	40555561	C	T	0.130	0.029	0.005	2.76E-08	632802	0.021	0.010	0.037
rs11872397	18	72535282	A	G	0.252	-0.025	0.004	1.43E-09	632802	-0.002	0.008	0.847
rs12025237	1	1.54E+08	C	A	0.127	-0.033	0.005	6.52E-10	632802	-0.006	0.010	0.529
rs12042107	1	91196176	C	T	0.543	-0.022	0.004	4.22E-10	632802	0.004	0.007	0.592
rs12112638	7	69735251	G	A	0.266	-0.025	0.004	1.34E-09	632802	-0.006	0.008	0.463
rs12186738	5	1.04E+08	T	G	0.157	-0.033	0.005	3.42E-11	632802	-0.002	0.009	0.847
rs12333760	7	99185406	C	T	0.163	-0.029	0.005	1.44E-09	632802	0.007	0.009	0.437
rs12356821	10	1.05E+08	C	G	0.146	0.039	0.005	6.27E-15	632802	-0.011	0.010	0.241
rs12441907	15	83922387	A	C	0.191	-0.029	0.005	1.06E-10	632802	0.021	0.008	0.012
rs12474587	2	1.63E+08	T	G	0.440	0.028	0.004	1.25E-14	632802	0.006	0.007	0.358
rs12545053	8	65073605	G	A	0.391	0.020	0.004	2.43E-08	632802	-0.003	0.007	0.709
rs12632110	3	50224225	G	A	0.650	-0.023	0.004	4.78E-10	632802	0.004	0.007	0.531
rs13030994	2	1.46E+08	A	G	0.493	0.036	0.004	3.56E-24	632802	-0.007	0.007	0.265
rs13145728	4	1.41E+08	C	G	0.382	-0.023	0.004	2.14E-10	632802	0.000	0.007	0.981
rs13261666	8	59814666	T	G	0.507	-0.027	0.004	3.90E-14	632802	0.007	0.007	0.310
rs134529	22	28781758	C	T	0.388	-0.020	0.004	4.85E-08	632802	0.002	0.007	0.815
rs1385108	5	1.55E+08	T	C	0.242	0.025	0.004	3.00E-09	632802	0.012	0.008	0.122
rs1435741	15	47935843	A	G	0.437	0.029	0.004	2.64E-16	632802	0.010	0.007	0.158
rs1445649	2	1.56E+08	C	T	0.529	0.024	0.004	1.68E-11	632802	0.005	0.007	0.490
rs1555445	20	31175258	T	A	0.327	0.023	0.004	3.65E-09	632802	-0.004	0.007	0.618
rs1565735	8	27426077	A	T	0.200	-0.038	0.004	3.42E-17	632802	-0.003	0.008	0.709
rs1869243	3	5724536	C	T	0.463	0.020	0.004	2.97E-08	632802	0.011	0.007	0.112
rs1899896	8	93201036	T	C	0.308	0.026	0.004	1.04E-11	632802	-0.003	0.007	0.715
rs1971318	12	1.21E+08	T	C	0.153	0.029	0.005	7.06E-09	632802	-0.017	0.009	0.068
rs2046850	1	2.1E+08	T	C	0.192	-0.025	0.004	3.03E-08	632802	-0.019	0.008	0.021
rs2050586	1	87915254	C	G	0.362	-0.021	0.004	3.00E-08	632802	0.017	0.007	0.016
rs2107300	2	2.01E+08	G	C	0.829	-0.027	0.005	3.27E-08	632802	-0.032	0.009	0.001
rs2140114	7	3407568	T	C	0.518	-0.023	0.004	4.70E-10	632802	-0.010	0.007	0.116
rs222449	6	52916062	T	A	0.784	-0.025	0.004	1.08E-08	632802	-0.003	0.008	0.736
rs2378662	9	86707289	A	G	0.534	0.021	0.004	4.16E-09	632802	0.009	0.007	0.203
rs240963	6	1.12E+08	C	T	0.821	-0.041	0.005	2.16E-17	632802	0.009	0.009	0.345
rs2631024	8	91995577	G	A	0.715	-0.023	0.004	1.18E-08	632802	0.009	0.007	0.216
rs266047	2	1.04E+08	A	G	0.532	-0.031	0.004	3.36E-16	632802	-0.016	0.007	0.016
rs3001723	1	44037685	A	G	0.293	0.034	0.004	8.12E-18	632802	0.010	0.007	0.152
rs301805	1	8481016	G	T	0.580	0.021	0.004	2.80E-09	632802	0.007	0.007	0.270
rs35702515	2	1.38E+08	T	G	0.238	0.025	0.004	2.43E-09	632802	0.012	0.008	0.158
rs3800227	6	1.09E+08	G	A	0.732	0.023	0.004	1.93E-08	632802	-0.011	0.008	0.145
rs3801289	7	96638267	C	A	0.348	-0.022	0.004	3.74E-09	632802	0.003	0.007	0.726
rs3904512	13	38357471	A	G	0.449	-0.021	0.004	3.23E-09	632802	-0.002	0.007	0.827
rs4044321	5	1.67E+08	G	A	0.634	-0.028	0.004	6.08E-14	632802	-0.006	0.007	0.372
rs4236259	7	1708080	G	T	0.491	-0.025	0.004	3.35E-12	632802	0.001	0.007	0.939
rs4352629	5	87756821	T	C	0.458	-0.028	0.004	1.22E-14	632802	-0.010	0.007	0.153
rs4523689	11	7950797	G	A	0.386	-0.021	0.004	1.55E-08	632802	-0.005	0.007	0.506
rs4543592	9	3014254	C	T	0.469	0.022	0.004	7.46E-10	632802	0.006	0.007	0.388
rs4674993	2	2.26E+08	G	A	0.203	-0.025	0.004	1.32E-08	632802	-0.006	0.008	0.480
rs4759228	12	56508409	C	G	0.287	-0.022	0.004	3.58E-08	632802	-0.010	0.008	0.197

rs4781977	16	17572674	C	T	0.206	-0.024	0.004	4.54E-08	632802	-0.003	0.008	0.739
rs4785836	16	65604652	C	T	0.382	-0.020	0.004	2.26E-08	632802	0.004	0.007	0.612
rs6265	11	27679916	T	C	0.182	-0.032	0.005	3.77E-12	632802	-0.004	0.009	0.608
rs6433897	2	1.82E+08	C	T	0.729	0.022	0.004	3.16E-08	632802	0.003	0.008	0.657
rs66680800	3	85985324	T	G	0.395	-0.020	0.004	2.83E-08	632802	-0.009	0.007	0.174
rs6669839	1	50625979	T	C	0.209	0.026	0.004	3.36E-09	632802	0.015	0.008	0.074
rs6728726	2	623976	C	T	0.811	0.035	0.005	6.73E-14	632802	0.037	0.009	0.000
rs6788098	3	85624131	T	A	0.619	-0.031	0.004	1.91E-17	632802	-0.019	0.007	0.005
rs6893752	5	60374912	G	A	0.732	-0.024	0.004	3.25E-09	632802	0.011	0.008	0.148
rs7197072	16	717085	T	C	0.248	-0.025	0.004	2.77E-09	632802	0.024	0.008	0.002
rs7224742	17	30657058	T	C	0.616	-0.021	0.004	1.43E-08	632802	0.008	0.007	0.231
rs72789632	5	1.07E+08	T	C	0.131	-0.033	0.005	5.02E-10	632802	0.002	0.010	0.875
rs72896886	18	42632652	C	G	0.158	-0.027	0.005	2.75E-08	632802	0.003	0.009	0.769
rs7322872	13	1.01E+08	T	C	0.761	-0.026	0.004	3.58E-09	632802	-0.017	0.008	0.041
rs7555507	1	73766037	T	C	0.504	-0.024	0.004	1.14E-11	632802	-0.007	0.007	0.289
rs76214862	14	29500130	C	A	0.187	-0.025	0.005	3.99E-08	632802	0.012	0.008	0.161
rs76608582	19	4474725	A	C	0.052	-0.050	0.008	1.94E-09	632802	-0.020	0.018	0.268
rs7929518	11	85980958	G	A	0.762	0.024	0.004	1.56E-08	632802	0.006	0.008	0.473
rs7938812	11	1.13E+08	G	T	0.384	0.044	0.004	2.71E-33	632802	0.003	0.007	0.707
rs7969559	12	69655167	G	A	0.710	-0.024	0.004	7.31E-10	632802	-0.001	0.007	0.852
rs9401770	6	98748008	A	G	0.277	0.028	0.004	3.47E-12	632802	-0.010	0.007	0.187
rs9423279	10	1.26E+08	G	C	0.634	-0.021	0.004	3.21E-08	632802	0.003	0.008	0.686
rs962625	4	28473524	G	A	0.265	0.024	0.004	4.37E-09	632802	-0.002	0.008	0.752
rs9835772	3	85766025	T	A	0.253	0.024	0.004	6.32E-09	632802	0.004	0.008	0.623
rs993700	4	67825894	C	T	0.762	-0.026	0.004	1.53E-09	632802	-0.016	0.008	0.043

SNPs, single nucleotide polymorphisms; EAF, effect allele frequency; SE, standard error.

Supplementary Table 2 Summary statistics for the instrumental variables associated with atrial fibrillation and smoking initiation, IEU OpenGWAS 2018-2019 (N=372,249)

SNPs	chromosome	position	effect allele	other allele	EAF	atrial fibrillation				smoking initiation		
						beta	SE	p	sample size	beta	SE	p
rs10141892	14	35184323	C	T	0.596	-0.045	0.007	2.95E-11	1030836	-0.007	0.004	0.055
rs10213171	4	1.49E+08	G	C	0.072	0.091	0.013	1.32E-11	1030836	0.004	0.007	0.628
rs10458662	10	77942661	G	T	0.166	0.054	0.009	6.93E-10	1030836	0.000	0.005	0.965
rs10520002	5	1.28E+08	A	G	0.091	0.063	0.011	2.85E-08	1030836	-0.005	0.006	0.359
rs10520260	4	1.74E+08	G	A	0.294	-0.046	0.007	3.36E-10	1030836	-0.006	0.004	0.106
rs10753933	1	2.03E+08	G	T	0.546	-0.061	0.007	9.84E-20	1030836	0.005	0.004	0.174
rs10773657	12	1.23E+08	A	C	0.869	-0.058	0.010	2.54E-08	1030836	-0.001	0.006	0.828
rs10804493	3	1.12E+08	A	G	0.653	0.056	0.007	1.63E-15	1030836	0.001	0.004	0.785
rs10821415	9	97723266	A	C	0.419	0.082	0.007	2.92E-34	1030836	0.000	0.004	0.933
rs10842383	12	24773919	T	C	0.144	-0.099	0.010	2.88E-25	1030836	-0.010	0.005	0.050
rs11191116	10	1.04E+08	T	C	0.329	-0.041	0.007	4.42E-09	1030836	0.013	0.004	0.000
rs11264280	1	1.55E+08	T	C	0.295	0.135	0.007	3.07E-79	1030836	0.010	0.004	0.008
rs11598047	10	1.05E+08	G	A	0.169	0.154	0.009	8.95E-66	1030836	-0.010	0.005	0.044
rs11773845	7	1.16E+08	A	C	0.592	0.105	0.007	2.39E-55	1030836	0.003	0.004	0.506
rs117984853	6	1.49E+08	T	G	0.080	0.123	0.012	1.34E-24	1030836	0.010	0.006	0.115
rs12245149	10	65321147	A	C	0.475	-0.047	0.007	1.66E-12	1030836	0.004	0.004	0.242
rs12426679	12	76237987	T	C	0.485	-0.039	0.007	4.95E-09	1030836	0.002	0.004	0.561
rs1278493	3	1.36E+08	A	G	0.543	-0.039	0.007	8.77E-09	1030836	-0.005	0.004	0.165
rs133885	22	26159289	A	G	0.466	0.041	0.007	2.22E-09	1030836	0.001	0.004	0.784
rs140185678	16	2003016	A	G	0.028	0.166	0.022	2.43E-14	1030836	-0.002	0.010	0.874
rs1458038	4	81182554	T	C	0.278	0.043	0.007	1.74E-09	1030836	0.006	0.004	0.129
rs146518726	1	51535039	A	G	0.022	0.161	0.021	8.27E-15	1030836	-0.015	0.011	0.173
rs1563304	17	44874453	T	C	0.157	0.064	0.009	2.56E-12	1030836	-0.010	0.005	0.040
rs17171711	5	1.37E+08	T	C	0.165	0.109	0.009	1.95E-35	1030836	0.011	0.005	0.017
rs17380837	12	26345526	T	C	0.288	-0.050	0.007	4.8E-12	1030836	-0.010	0.004	0.011
rs1838747	5	1.14E+08	G	A	0.488	0.039	0.007	4.13E-09	1030836	-0.011	0.004	0.002
rs1906615	4	1.12E+08	T	G	0.235	0.366	0.008	1E-200	1030836	0.006	0.004	0.156
rs2031522	6	87821501	G	A	0.384	-0.044	0.007	1.47E-10	1030836	-0.001	0.004	0.855
rs2274115	9	1.39E+08	G	A	0.692	0.049	0.008	1.69E-10	1030836	-0.002	0.004	0.657
rs2288327	2	1.79E+08	G	A	0.175	0.092	0.009	7.26E-25	1030836	-0.009	0.005	0.068
rs2359171	16	73053022	A	T	0.176	0.175	0.009	4.65E-91	1030836	-0.008	0.005	0.106
rs2540949	2	65284231	T	A	0.387	-0.066	0.007	2.95E-22	1030836	0.002	0.004	0.602
rs2738413	14	64679960	G	A	0.533	-0.078	0.007	2.55E-31	1030836	0.001	0.004	0.882
rs2739197	4	1.12E+08	G	C	0.259	0.116	0.009	3.2E-41	1030836	-0.003	0.004	0.489
rs2759301	15	80994288	A	G	0.427	0.039	0.007	5.04E-09	1030836	0.011	0.004	0.002
rs2834618	21	36119111	G	T	0.111	-0.094	0.011	3.41E-17	1030836	-0.001	0.006	0.908
rs28387148	2	1.27E+08	T	C	0.099	0.074	0.011	6.25E-11	1030836	-0.004	0.006	0.508
rs284277	1	10790797	A	C	0.621	-0.042	0.007	1.25E-09	1030836	-0.004	0.004	0.237
rs2860482	12	57105938	C	A	0.733	-0.054	0.008	1.21E-12	1030836	0.000	0.004	0.968
rs28631169	14	23888183	T	C	0.178	0.052	0.008	5.35E-10	1030836	0.002	0.004	0.602
rs2885697	1	41544279	T	G	0.678	-0.044	0.007	2.88E-10	1030836	0.003	0.004	0.378
rs3176326	6	36647289	A	G	0.209	-0.063	0.009	1.42E-13	1030836	-0.004	0.004	0.405
rs337705	5	1.14E+08	G	T	0.407	0.056	0.007	1.63E-16	1030836	-0.007	0.004	0.045
rs34080181	3	66454191	A	G	0.368	-0.045	0.007	1.28E-10	1030836	-0.008	0.004	0.034
rs34936990	10	1.06E+08	A	G	0.119	0.129	0.010	2.95E-37	1030836	-0.003	0.006	0.578
rs34969716	6	18210109	A	G	0.290	0.070	0.008	1.6E-19	1030836	-0.007	0.004	0.080
rs35544454	2	2.13E+08	T	A	0.176	-0.059	0.009	1.1E-11	1030836	0.000	0.005	0.962
rs35569628	13	1.14E+08	C	T	0.237	-0.045	0.008	1.38E-08	1030836	0.000	0.004	0.993
rs35963991	8	11495702	T	G	0.155	0.053	0.010	2.8E-08	1030836	-0.006	0.005	0.197
rs3943207	8	21845619	T	G	0.136	-0.064	0.010	6.92E-10	1030836	-0.007	0.006	0.224
rs4073778	1	1.16E+08	A	C	0.554	0.049	0.007	4.96E-13	1030836	-0.002	0.004	0.583
rs4252627	17	37868715	T	C	0.646	-0.042	0.007	5.63E-09	1030836	-0.015	0.004	0.000
rs4587869	14	32992334	C	G	0.290	0.072	0.008	1.19E-20	1030836	0.002	0.004	0.691
rs4642101	3	12842223	G	T	0.641	0.071	0.007	2.95E-24	1030836	0.003	0.004	0.357
rs464901	22	18597502	C	T	0.346	-0.051	0.007	1.53E-12	1030836	0.002	0.004	0.683
rs4757877	11	20010291	G	A	0.765	-0.072	0.008	2.93E-20	1030836	0.005	0.004	0.253
rs4935786	11	1.22E+08	A	T	0.712	-0.046	0.008	4.85E-09	1030836	0.016	0.004	0.000
rs4946333	6	1.19E+08	G	A	0.510	0.064	0.007	5.47E-22	1030836	-0.001	0.004	0.869
rs4965430	15	99268850	G	C	0.607	-0.044	0.007	1.26E-10	1030836	-0.008	0.004	0.032
rs55734480	7	14372009	A	G	0.254	0.055	0.008	2.2E-12	1030836	-0.001	0.004	0.717

rs55985730	7	1.28E+08	G	T	0.049	0.087	0.015	5.24E-09	1030836	0.002	0.008	0.802
rs56201652	7	92278116	A	G	0.286	-0.053	0.008	1.74E-12	1030836	0.009	0.004	0.030
rs56326533	2	2.01E+08	C	T	0.390	0.069	0.007	6.28E-24	1030836	0.004	0.004	0.288
rs577676	1	1.71E+08	T	C	0.458	-0.092	0.007	1.62E-43	1030836	-0.002	0.004	0.495
rs60902112	3	1.95E+08	T	C	0.224	0.045	0.008	1.72E-08	1030836	0.001	0.004	0.874
rs62254082	3	69417585	C	T	0.355	0.040	0.007	6.34E-09	1030836	0.005	0.004	0.139
rs62377206	5	1.68E+08	A	G	0.050	0.085	0.015	8.21E-09	1030836	-0.010	0.008	0.195
rs62521286	8	1.25E+08	G	A	0.058	0.120	0.014	4.5E-19	1030836	0.005	0.007	0.463
rs6462079	7	28415827	A	G	0.764	0.047	0.008	8.79E-10	1030836	0.004	0.004	0.308
rs6546620	2	26159940	C	T	0.760	0.060	0.009	3.19E-12	1030836	0.000	0.005	0.928
rs6560886	12	1.33E+08	C	T	0.821	0.051	0.009	1.49E-08	1030836	-0.003	0.004	0.505
rs6580277	5	1.43E+08	G	A	0.224	0.067	0.008	1.64E-17	1030836	0.001	0.004	0.897
rs6596717	5	1.06E+08	A	C	0.615	-0.040	0.007	3E-09	1030836	0.014	0.004	0.000
rs6689306	1	1.54E+08	G	A	0.562	-0.046	0.007	1.36E-11	1030836	-0.002	0.004	0.619
rs6747542	2	70106832	C	T	0.468	-0.055	0.007	1.1E-16	1030836	0.002	0.004	0.618
rs6771054	3	89523038	C	T	0.381	-0.046	0.007	2.42E-11	1030836	0.011	0.004	0.002
rs6790396	3	38771925	G	C	0.591	0.063	0.007	2.4E-20	1030836	-0.002	0.004	0.670
rs67969609	2	1.46E+08	G	C	0.089	0.071	0.013	1.71E-08	1030836	-0.001	0.007	0.936
rs6838973	4	1.12E+08	T	C	0.435	-0.151	0.007	1E-111	1030836	0.004	0.004	0.288
rs6882776	5	1.73E+08	A	G	0.318	-0.071	0.007	9.64E-22	1030836	0.000	0.004	0.974
rs6994744	8	1.42E+08	C	A	0.489	0.041	0.007	1.09E-09	1030836	-0.012	0.004	0.001
rs71454237	12	70013415	A	G	0.209	-0.062	0.008	1.78E-13	1030836	0.001	0.004	0.818
rs7172038	15	73667255	G	T	0.163	0.112	0.009	4.78E-36	1030836	0.001	0.005	0.858
rs7224711	17	76772288	T	C	0.529	-0.037	0.007	3.72E-08	1030836	0.007	0.004	0.049
rs7225165	17	1309850	A	G	0.112	-0.066	0.011	3.2E-09	1030836	-0.007	0.006	0.220
rs72694603	1	1.12E+08	T	C	0.291	-0.055	0.007	2.26E-14	1030836	-0.005	0.004	0.177
rs72700114	1	1.7E+08	C	G	0.064	0.202	0.013	3.29E-54	1030836	0.009	0.007	0.208
rs72811294	17	12618680	C	G	0.115	-0.072	0.011	9.67E-12	1030836	0.000	0.006	0.957
rs72926475	2	86594487	A	G	0.122	-0.068	0.010	2.37E-11	1030836	0.002	0.005	0.728
rs72966339	6	1.22E+08	T	C	0.365	-0.062	0.007	7.42E-19	1030836	0.000	0.004	0.922
rs73041705	3	24463235	C	T	0.299	-0.044	0.007	1.55E-09	1030836	-0.004	0.004	0.326
rs73366713	6	16415751	A	G	0.132	-0.104	0.010	1.53E-25	1030836	-0.002	0.005	0.718
rs74500426	4	1.75E+08	T	G	0.086	-0.092	0.013	4.29E-13	1030836	-0.001	0.007	0.918
rs74832855	1	1.7E+08	G	A	0.035	0.122	0.018	1.43E-11	1030836	0.012	0.010	0.221
rs74884082	14	73249419	T	C	0.249	-0.049	0.008	3.48E-10	1030836	-0.004	0.004	0.302
rs74910854	7	74110705	G	A	0.072	0.090	0.016	4.31E-08	1030836	-0.004	0.007	0.636
rs7508	8	17913970	A	G	0.724	0.071	0.008	1.69E-21	1030836	0.010	0.004	0.011
rs7529220	1	22282619	C	T	0.849	0.062	0.010	1.98E-10	1030836	-0.009	0.005	0.087
rs7574892	2	1.76E+08	A	G	0.465	0.055	0.007	1.98E-16	1030836	0.002	0.004	0.599
rs76097649	11	1.29E+08	A	G	0.082	0.115	0.012	1.26E-20	1030836	0.007	0.006	0.294
rs7612445	3	1.79E+08	T	G	0.204	0.049	0.008	4.81E-09	1030836	0.001	0.005	0.832
rs775498	12	70071513	G	A	0.299	0.042	0.007	1.05E-08	1030836	0.009	0.004	0.016
rs7789146	7	1.51E+08	A	G	0.192	-0.058	0.009	2.12E-11	1030836	0.005	0.005	0.324
rs7915134	10	75420570	T	C	0.151	-0.117	0.010	1.42E-34	1030836	-0.004	0.005	0.464
rs79187193	1	1.47E+08	A	G	0.040	-0.116	0.015	3.15E-14	1030836	-0.001	0.008	0.911
rs8088085	18	48708548	C	A	0.442	-0.037	0.007	4.79E-08	1030836	0.002	0.004	0.593
rs883079	12	1.15E+08	T	C	0.721	0.098	0.007	2.84E-40	1030836	-0.003	0.004	0.474
rs9506925	13	23368943	T	C	0.268	0.045	0.008	2.72E-09	1030836	0.002	0.004	0.693
rs9953366	18	46474192	C	T	0.683	0.049	0.007	1.82E-11	1030836	-0.005	0.004	0.176

SNPs, single nucleotide polymorphisms; EAF, effect allele frequency; SE, standard error.

© 2024 Wu Z. et al.
